# The Diet of Children Attending a Holiday Programme in the UK: Adherence to UK Food-Based Dietary Guidelines and School Food Standards

**DOI:** 10.3390/ijerph19010055

**Published:** 2021-12-22

**Authors:** Eilish Crilley, Iain Brownlee, Margaret Anne Defeyter

**Affiliations:** Healthy Living Lab, Faculty of Health and Life Sciences, Northumbria University, Newcastle upon Tyne NE1 8ST, UK; iain.brownlee@northumbria.ac.uk (I.B.); greta.defeyter@northumbria.ac.uk (M.A.D.)

**Keywords:** diet quality, nutrition, holiday programme, Eatwell guide, school food standards

## Abstract

Child poverty rates are rising, particularly in London, putting more children at risk of experiencing food insecurity. Holiday programmes in the UK provide children who receive free schools meals during term time with access to free/low-cost holiday clubs offering nutritious food and enriching activities during the school holidays. This study aimed to investigate whether children’s dietary intake was more adherent to the UK Eatwell Guide throughout the day and meets School Food Standards (SFS) for the lunchtime meal on a club attendance versus a non-attendance day. A repeated measures design was used to assess data on the food and drink intake of children (*n* = 57) aged 7–16 years old using a 24 h recall method on two separate occasions: once based on an attending club day and once based on a non-attending club day. The results showed children’s diet quality improved (*p* = 0.007) on an attending club day (mean: 58.0 ± SD 12.6) versus a non-attending club day (51.8 ± 15.0). Children also more closely adhered to the SFS (*p* = 0.001) on an attending club day (median = 9, interquartile range = 8–9) versus a non-attending club day (median = 7, interquartile range = 6–8). This suggests that holiday programmes targeting children who receive free school meals during term time have the potential to improve children’s dietary behaviours during the school holidays, underlining the importance of holiday programmes to support food security.

## 1. Introduction 

Food insecurity in the UK is among the highest in Europe [1]. A recent report [2] states that levels of food insecurity are particularly high in London with 400,000 children under 16 years old struggling to afford or access enough food. This is unsurprising, as recent reports show that child poverty rates in London are among the highest in the UK with four in ten children living in poverty in the city [3,4]. High child poverty rates in the city are particularly relevant as the holiday clubs involved in the current study are part of the Kitchen Social holiday programme, which operates across London and targets families in receipt of Free School Meals.

The nutritional benefits children may receive from school food programmes and breakfast clubs during the school term are not accessible during the school holidays and food intake during the school holidays would be expected to negatively differ and be the least ideal. Yet surprisingly, few studies have investigated children’s food/drink intake during the school holidays. The only UK study (known to date) that focused on term time and school holiday food/drink consumption found no differences in children’s energy and fat intake during these time periods [5]. However, this study [5] was carried out over 10 years ago, and since then, children’s diets may have changed. The method also relied upon Australian healthy eating guidelines to categorise foods/drink as healthy and not healthy rather than using the UK Eatwell Guidelines.

In the UK, there has been a growth in holiday clubs set up to support children and families at risk of food insecurity. Research suggests that holiday clubs are successful in mitigating food insecurity, as food insecure households were found to benefit more from holiday clubs compared to food secure households [6]. These holiday clubs have the intention of providing children with nutritious food to replace their free school meal, alongside activities, during the school holidays [7,8]. However, whilst holiday clubs are advised to comply with the School Food Standards in England, there is no clear accountability mechanism [9]. Despite the provision of food at clubs to support families experiencing food insecurity and the intention of clubs to provide children with nutritious foods, there are a lack of research studies investigating the impacts of holiday clubs on children’s nutritional intake. The only published research studies that have investigated children’s diets based on holiday club attendance includes an evaluation of “A Day Out, Not a Handout” holiday programme across the North East and a published study examining the “Food and Fun” holiday clubs in Wales [10,11]. However, these studies did not collect data at a macronutrient level.

Assessing children’s overall diet is essential because the relationship between dietary intake and health is very complex and cannot be captured by studying single dietary components [12]. Diet quality refers to a priori scores or indices that are used to assess how well an individual’s diet agrees with a predetermined notional ideal of dietary intake [13]. In the UK, the Eatwell Guidelines are promoted as a guide for a healthy diet. This guide defines a healthy diet as at least five portions of fruit and vegetables daily and food and drinks of lower fat, salt, and sugar from three main groups (starchy foods, protein sources, and dairy or alternatives). The guide also recommends consuming six to eight glasses of water daily and reducing sugar-sweetened beverages, processed meat, and red meat intake. In government-funded schools in England, the School Food Standards define minimal nutritional standards to ensure children are provided with nutritious meals. To meet these standards, school caterers are provided with advice on the types of foods to serve and how much to serve. For example, one or more portions of fruit and vegetables should be served each day. These standards aim to help children to develop healthy eating habits [14].

Although non-Holiday Activity and Food (HAF) programme-funded holiday clubs do not have to comply to any specific regulations [15], the majority of non-HAF funded holiday clubs and all clubs funded by the HAF programme (Department for Education) [16] are encouraged to follow the School Food Standards. The holiday clubs involved in the Kitchen Social holiday programme included in this current study are also required to adhere to School Food Standards [17]. Yet, similar to some of the HAF-funded clubs, some of the clubs involved in this study provide open access provision where children can access food off-site. Therefore, it is unknown whether children are consuming the food provided at the club and/or whether they rely more on purchasing food to eat from nearby shops. 

Consuming a diet that meets the UK’s Eatwell Guide recommendations requires a higher proportion of disposable income [18]. Therefore, positive food choices are clearly less affordable to low-income households [19], and this may result in children purchasing cheaper, less healthy food. Alternatively, if children do consume the food provided at the club, it is unknown whether this makes any difference to their overall dietary intake. There have been limited attempts to evaluate food provision in holiday clubs (as discussed earlier). Therefore, due to these gaps in the literature, the aims of this study are as follows:

1. To investigate whether children’s dietary habits throughout the day were more adherent to the UK Eatwell Guide on a club attendance day versus a non-attendance day.
2. To investigate whether children’s food and drink intake meets School Food Standards (SFS) in a holiday club meal versus a comparable meal outside of holiday clubs.

The following objectives were set in order to fulfil these aims:

1. Collect and analyse 24 h dietary recall data from holiday club users on attendance and non-attendance days.
2. Compare children’s dietary intake on attending and non-attending days to national food standards.

## 2. Materials and Methods

### 2.1. Study Design

This quasi-experimental study (i.e., an empirical interventional study used to estimate the causal impact of an intervention on the target population without random assignment) utilised a repeated measures design: a day attending club versus a non-attending club day. The dependent measures were (1) diet quality in relation to UK food-based dietary guidelines and (2) adherence of the lunch meal to School Food Standards on an attending versus a non-attending club day. Due to limited potential to collect data from children, data on club attendance days were collected for a weekday, whereas the non-club attendance days were collected for a weekend day.

### 2.2. Participants 

Ethical approval (Approval number: 16405) was gained from the Faculty of Health and Life Science’s Ethics Committee at Northumbria University on 20 July 2019. A non-probability purposive sampling strategy was used in the current study as the researcher recruited children who met the study criteria of attending the Kitchen Social holiday programme. In total, 63 children were recruited from 5 holiday clubs participating in the Kitchen Social holiday programme. Due to sporadic attendance rates, 6 children recorded their food intake at only one time point, and these children did not attend again during the remainder of the testing visits conducted by the researcher. Therefore, a second recording of food intake could not be followed up with these participants. The remaining sample of 57 children (33 females and 24 males) recorded their food intake at both time points. The age range included in this study of 7–16 years old (mean age: 10.8, standard deviation: 2.0) was used, as this was the target population of the clubs that consented to participate in this study, and research states that children aged 7+ are cognitively capable of retrospectively recalling their food and drink intake [20].

### 2.3. Procedure

Kitchen Social co-ordinators contacted all of the holiday club leaders participating in their programme and invited them to participate in this study. Club leaders who expressed interest in participating in the study were contacted by the researcher and were provided with further details of the study and a consent form. Overall, 5 clubs consented to take part in the study. The holiday programme involved in this study delivered holiday clubs in a range of different venues, including outdoor adventure playgrounds and community centres. All the holiday clubs were located in areas of high deprivation serving families who are normally in receipt of Free School Meals during term time. However, the current study did not measure individual household income. Further information on the holiday clubs involved in this study are provided in Table 1. Once the researcher had received written consent from the club leaders, parents received study information and consent forms. Then, the researcher arranged dates and times to visit each club to recruit child participants. During the research visits at the clubs, the researcher approached each child whose parents had provided fully informed consent and verbally informed each child of the research study. Then, those children who were interested in taking part were given an information and consent form that provided them with further details of the study. Nutritional data were collected via a retrospective 24 h recall. The Young Persons Food Atlas (YPFA) assisted children in estimating portion sizes and cooking brands during the 24 h recall, allowing the researcher to collect more accurate detail on dietary intake. The YPFA is a booklet developed by Foster and Adamson (2012) [21] that contains child-friendly photographs of various portion sizes of different foods and drinks. This is a validated tool and has been shown to be as accurate for parents’ food/drink recall for children aged 11–16 years old [21,22]. This tool has also been used in younger age groups, from 4 to 11 years, with results showing that children’s portion size estimates were more accurate using age-appropriate food/drink photographs compared to when children were using photographs of adult’s portion sizes [23]. On both data collection days, children were offered a sticker as a token of appreciation. Children were also provided with a debrief form upon completion of the study.

### 2.4. Adherence of Daily Dietary Intake to Food-Based Dietary Guidelines

To achieve the aims of the current study, 24 h recall data on attendance and non-attendance days were collected. Due to limited opportunities to collect data during a weekday on the non-attending day, children’s nutritional intake was collected on a weekday when they attended the hub versus a weekend day when they did not attend the hub.

The YPFA was used during 24 h recalls to support children in their estimation of the portion sizes they consumed in grams (including information on leftovers). To study children’s overall diet from data on all of the meals children consumed on an attending versus a non-attending day, the current study used researcher-defined scores of diet quality, which are based on guidelines for a healthy diet [12]. More specifically, UK Eatwell Guidelines [24] were used to evaluate children’s diet quality on a day they attended a holiday club compared to a non-attending day.

Eleven categories were developed to assess children’s diet quality out of a score of 110. Equal weighting was given to each diet quality category with a higher score reflecting greater compliance to the Eatwell Guide. To get a score of 10 in one of the categories, the child must have met or surpassed the recommendation from the Eatwell Guidelines. Seven of the categories were based on children’s nutritional intake, whereas four of the categories were based on the actual foods and drinks consumed throughout the day. See Table 2 for further details of category scoring criteria. Microdiet version 2.8 dietary analysis software (developed by the University of Salford in Manchester, UK), and additional detail from McCance and Widdowson’s ‘composition of foods integrated dataset’ and ‘composition of old foods’ [25] were utilised to estimate energy and nutrient intake. Scoring from 0 to 10 was continuous, and children could get any score from 0.0 to 10 (for example, a child could have a final diet quality score of 7.4). This method is based on previous research that has focused on the diet quality of children in Singapore using food-based dietary guidelines of the Singapore Health Promotion Board [26].

### 2.5. School Food Standards

In addition to comparing children’s entire daily intake to the Eatwell Guide, the food/drink that children consumed for lunch were compared to the School Food Standards. The meal children consumed for lunch was of specific interest, as this meal served as a replacement for the free school meal that children would have received during term time. As only one day of food intake at the holiday club was recorded, weekly recommendations were not included in this scoring (e.g., oily fish once or more every three weeks). Each recommendation in the School Food Standards (i.e., one or more portions of fruit every day) identified in Table 3 was scored 1, and children received a total score out of 12. A higher score demonstrated higher adherence of their lunchtime meal to the School Food Standards. The scoring categories from the School Food Standards which were used are presented in Table 3.

### 2.6. Dietary Analysis 

Then, data on children’s diet quality were entered into IBM SPSS Statistics 25 (IBM Corporation, New York, NY, USA) and analysed using Paired Samples *t*-tests and Wilcoxon Signed-Rank Tests. Analyses of children’s overall diet quality score and fruit and vegetable category score on an attending versus non-attending club day were conducted using a Paired Samples *t*-test. The Wilcoxon Signed-Rank Test was used to analyse individual diet quality categories (excluding the fruit and vegetable category, which was analysed using a Paired Samples *t*-test) and to compare children’s food and drink consumption for lunch on an attending and non-attending day to the School Food Standards. The McNemar test was also used to determine whether there were any differences in the dichotomous dependent variables (the percentage of children either scoring/not scoring maximally for each diet quality category or meeting/not meeting each element of the School Food Standards).

## 3. Results

### 3.1. Total and Individual Diet Quality Scores

There was a significant effect of club attendance (*p* = 0.007) on children’s diet quality score on an attending versus a non-attending day. On an attending day (mean: 58.0 ± standard deviation: 12.6), children have a higher diet quality score compared to a non-attending day (51.8 ± 15.0). 

**Figure 1:**
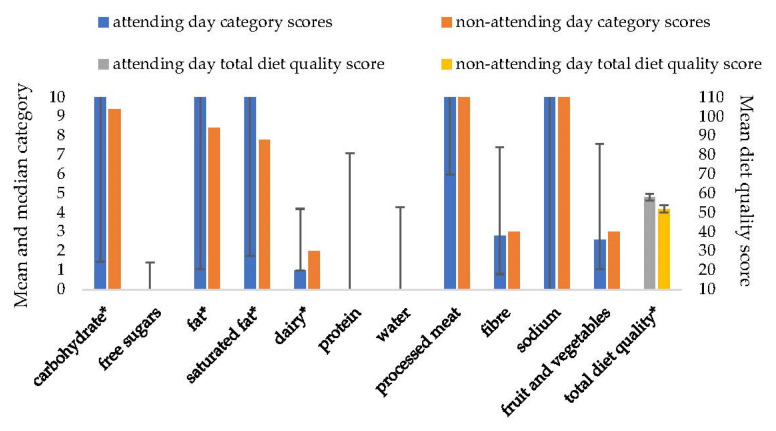
Mean (±SD) for total diet quality scores and mean (±SD) and median (IQR) for individual category scores across all participants (*n* = 57). Statistically significant differences (*p* < 0.05) are highlighted with *.

There was also a significant difference in carbohydrate (*p* = 0.002), fat (*p* = 0.001), and saturated fat (*p* = 0.001) category scores on an attending day (carbohydrate (median= 10.0, interquartile range = 8.55–10), fat (10.0, interquartile range = 8.95–10), and saturated fat (10.0, interquartile range = 8.25–10)) versus category scores on a non-attending day in carbohydrate (9.4, interquartile range = 0.95–10), fat (8.4, interquartile range = 3.15–10), and saturated fat (7.8, interquartile range = 0–10)). The median dairy category score (median = 1, 1.0, interquartile range = 0–3.2) was significantly lower on an attending day compared to the non-attending day (median = 2, 2.0, interquartile range = 0–3.3) (*p* = 0.045). See Figure 1 for further details.

Table 4 summarises the percentage of children meeting guidelines for category scores and total diet quality scores. A higher percentage of children scored maximum scores for most categories on days they were attending holiday clubs compared to days they were not attending holiday clubs. The categories with the lowest proportion of participants meeting dietary recommendations appeared to be dairy (0% across both attending and non-attending days), fibre (1.8% on both days), and protein (5.3% on attending days versus 0% on non-attending days). However, no children’s total dietary quality scores were at the ceiling. The McNemar test found a significantly higher proportion of children meeting the carbohydrate (*p* = 0.007), fat (*p* = 0.023) and saturated fat diet quality category (*p* = 0.001) on an attending versus a non-attending club day. No other category comparisons were statistically different.

### 3.2. School Food Standards 

There was a significant effect of attendance on total median School Food Standards scores (*p* = 0.001). Children had a higher School Food Standards median score (median = 9, interquartile range = 8–9) on an attending club day compared to a non-attending club day (median = 7, interquartile range = 6–8). Table 5 shows that a higher percentage of children met the recommendations for the School Food Standards on an attending versus a non-attending club day apart from for dairy and alternatives (where only 10.5% met the recommendation on both days) and healthier drink consumption (38.6% met the recommendation on attendance days versus 42.1% on non-attendance days). There was a significantly higher percentage of children meeting standards for starchy foods (*p* = 0.004), fruit and vegetables (*p* = 0.001), protein sources (*p* = 0.001), and discretionary foods (*p* = 0.001) consumption on an attending versus a non-attending club day. The proportions of individuals meeting other standards were not statistically different.

## 4. Discussion

This study highlighted that attendance at the Kitchen Social holiday programme appears to improve children’s overall dietary habit in comparison to dietary intake on non-attendance days. The data showing children are more likely to consume foods that better align with the School Food Standards on an attending versus a non-attending day suggests that the meals consumed at clubs are better than a comparable meal collected outside of clubs. Previous studies have suggested that holiday club attendance might result in meals being omitted during the rest of the day [15,28]. Our findings on improved adherence to food-based dietary guidelines suggest that overall dietary intake was also improved when attending clubs. The approach to estimate adherence to UK dietary guidelines in children and meal choice to School Food Standards is novel, and it is likely that these approaches could be applied to a wider range of dataset analysis and research questions. It is important to record children’s food and drink consumption across an entire day, as it is plausible that out-of-club meals may be modified as a result of children’s club attendance [15,28].

The results of the current study show that children have a better diet quality score on an attending club day compared to a non-attending club day, highlighting that children are more likely to adhere to the UK Eatwell Guidelines when they attend the club compared to days they do not attend. Greater adherence to the UK Eatwell Guidelines would be expected to bring numerous health benefits at a population level, including reduced prevalence to type 2 diabetes, lower rates of cardiovascular disease, and colorectal cancer and increased life expectancy [29]. 

The mean overall diet quality scores in the current study were generally low, suggesting that the dietary intake of the current cross-section of children is far from the notional ideal of the UK Eatwell Guidelines both on an attending and non-attending club day. Few children met most guidelines, as evidenced by infrequent maximal component scores (see Table 4). These findings broadly align with nationally representative data from the National Diet and Nutrition Survey (NDNS) over the 9–11-year period between 2008 and 2017/2019, which highlights that dietary guidelines are poorly adhered to nationally in both adults and children [30]. Similar results have also been found in other countries; for example, research focusing on children’s diet quality shows that these children have a low adherence to dietary guidelines, specifically for salt intake [26,31]. Low-income populations in the UK are even less likely to adhere to nutritional guidelines compared to the general population [32]. Therefore, the higher adherence to dietary guidelines on an attending club day compared to a non-attending club day may be particularly positive in supporting dietary intake in holiday club attendees outside of the school term. 

Previous research has suggested that children from low-income populations were more likely to consume diets that consist of high-fat and energy-dense foods [33]. Energy-dense meals are associated with poorer diet quality along with being positively associated with high fat intake [34], which suggests that high intakes of fat are associated with lower diet quality [35]. Research also suggests that children were more likely to consume a poor diet during the school summer holidays [36]. This has been supported in a research study by Grimes, Riddell, and Nowson [37], which found that on days children did not attend school, children’s diets consisted of higher levels of fat, saturated fat, and sugars compared to school days. However, these studies were carried out in the USA and Australia and may not be applicable to the UK. The current findings found that holiday club attendance supports improvements in dietary fat intake, ensuring that children meet daily fat recommendations. Similarly, median category scores for saturated fat and carbohydrate intake also improved as a factor of attendance despite a high proportion of children scoring maximally for these categories on both attendance and non-attendance days.

Dairy category scores were lower on an attending club day versus a non-attending club day in the current study, suggesting a need to explore approaches to increase dairy intake within the holiday club setting. Dairy products are important as they contain essential nutrients to ensure healthy growth and bone development in children [38,39]. Dairy products contain multiple nutrients, with total and low-fat dairy product consumption associated with a reduced risk of developing metabolic syndrome and a lower risk of dental caries and type 2 diabetes [38,40]. The median component scores for dairy intake suggest that few children in this cross-section were close to meeting the recommended three portions of dairy products on either attending or non-attending days. Similar findings to the current study have also been found in prior research studies, which show that a significant proportion of children in developed countries fail to consume the daily recommended intake of dairy products [38,39,40]. 

The current study findings also suggested there was no difference in children’s individual category scores for fruit and vegetable, free sugars, protein, water, processed meat, fibre, and sodium. These findings suggest that children require additional dietary intake support through club attendance. Although there was no difference in free sugar intake based on club attendance, the median scores presented in Figure 1 and percentages meeting the UK Eatwell Guidelines presented in Table 4 suggest that children did not meet free sugar dietary recommendations on either day, which aligns with data on the diets of the general population [30]. Unlike prior studies, the current study found no difference in children’s fruit and vegetable intake based on club attendance. To illustrate, a recent study [11] gathered survey data from children and found that on an attending club day, children self-reported that they consumed more fruits and vegetables compared to an non-attending day. However, this study [11] collected questionnaire data on dietary habit, and the data collected in the current study would be expected to more objectively and accurately represent dietary intake in the short-term [41]. 

The median scores highlighted in Figure 1 and the percentages of children meeting the UK Eatwell Guidelines in Table 4 suggest that a high percentage of children are not meeting dietary guidelines regardless of attendance. If almost no child meets minimal category scores on either day, this suggests that the problem is not specific to holiday clubs, but that there is a wider need to address the dietary intake of this population of children. Categories with the lowest diet quality scores include protein, water, and free sugar intake, suggesting the need for a tailored intervention aimed at increasing children’s consumption of these nutrients. However, despite children not fully adhering to the UK Eatwell Guidelines on either an attending or non-attending club day, children’s intake of fat, saturated fat, and carbohydrates improved as a result of holiday club attendance. Although mixed, the results suggest that holiday clubs may be an effective mechanism to improve children’s overall diet. 

Evaluation of the adherence of holiday clubs to School Food Standards is timely, as the DfE (Department for Education) has recently extended the funding of Holiday Activities and Food (HAF) programme across all 151 higher tier Local Authorities in England at a cost of £220 M, with a condition of funding being that food served in holiday clubs adheres to School Food Standards. The current study also enabled the comparison of lunches consumed in and out of holiday clubs. While the approach to data collection did not allow the consideration of all recommendations that make up the School Foods Standards, the findings clearly demonstrate that lunches consumed in holiday clubs were closer to meeting these standards than lunches consumed by attendees outside of holiday clubs. The current study finding partially supports prior qualitative reports by staff which state that holiday clubs adhere to school food standards [9,15]. However, the findings of the current study suggest that there is room for further improvement in the how holiday clubs (a) provide food that adheres to School Food Standards and (b) utilise tools to encourage children to engage in better dietary habits [7]. 

A limitation of the current study is that data on children’s nutritional intake was collected on a weekday when they attended the club versus a weekend day when they did not attend the club. Previous research has suggested that there are differences in children’s nutritional intake on a weekend compared to the weekday, with fat intake higher on the weekend and fruit and vegetable intake lower on the weekend [42,43]. However, these studies do not focus on school holiday periods and results appear to be rather mixed, with evidence from a prior study [5] showing no difference in daily, total, or saturated fat intake between weekdays and weekend days during the school term or the holiday periods. Nonetheless, the potential that the observed findings are a result of data collection at different timepoints in the week cannot be discounted.

A further important limitation of the current study to acknowledge is that the collection of dietary data over one day provides only a snapshot of general consumption patterns. Gathering food and drink intake data on one attending and one non-attending day provides a snapshot of dietary habit aligned to club attendance or non-attendance but may not be sufficient to account for habitual dietary intake, with at least two consecutive days of intake recommended [44].

The current study has a low participant sample; however, the researcher experienced significant difficulties in collecting data across the school holidays as children have sporadic attendance rates at the clubs. Whilst collecting dietary data for a longer time period would be preferable in future studies, this study has presented the most robust and reliable data (to date) on dietary consumption across the school holidays for children living in under-served communities. The comparisons carried out here provide a snapshot of change in dietary intake for individual attendees on days they attended or did not attend clubs. However, the current study design does not have a comparator group who have not signed up for and attended holiday clubs. The inclusion of such a group alongside holiday club attendees in the future would allow a more direct comparison of the impact of club attendance on dietary intake.

Due to the above limitations, the authors advise that the findings are interpreted cautiously. However, this is the first study (to the authors’ knowledge) that has focused on the type and amount of food that children consume across entire days during the school summer holidays and at holiday club. In the future, holiday club staff may wish to take note of strategies to increase children’s dairy intake, since intakes of dairy appear to be low on attendance and non-attendance days. Parents use a number of strategies to increase their children’s uptake of dairy products such as by ensuring the dairy product is visually appealing, available, and accessible for their child to consume [39]. The current study compared children’s dietary intake to the School Food Standards, yet these standards have not been used to evaluate the holiday club menus. Therefore, future studies should view whether the holiday clubs are providing food that adheres to the School Food Standards, and more importantly, if children are eating the food provided. Future studies may also consider collecting dietary intake data from parents, as previous research demonstrates that parents skip meals during the school holidays so that their children can eat [45].

## 5. Conclusions

In conclusion, children’s dietary intake appeared to be more positive on hub attendance days versus non-attendance days in a small cross-section of children. Yet hubs could still improve their food/drink offer, as children did not meet the UK Eatwell Guidelines for some groups included in the diet quality scoring, including fruit and vegetable intake. Additionally, children’s lunch time meals did not fully adhere to the School Food Standards. This suggests that the hubs require further support to provide nutritional meals to children.

## Figures and Tables

**Table 1:** Operational information on the holiday clubs involved in the current study. * Information from the English Index of Multiple Deprivation 2015, which ranks every small area in England from 1 (most deprived area) to 32,844 (least deprived area) based on a range of deprivation indicators including income, employment, health, education, crime, and living environment.

Type of Holiday Club	Meals Served	Daily Maximum Number of Children Attending	Opening Days	Opening Times	* Index of Multiple Deprivation Score (IMD)
1. Community centre	Lunch served	80 50 maximum attendances for food provision	Monday to Friday	10–4 pm	22,860
2. Community centre	Lunch served	190	Monday to Friday	10–5:30 pm	19,906
3. Adventure playground	Lunch served	No maximum (usually 40–50 children attend)	Monday to Friday	10–5 pm	1876
4. Adventure playground	Lunch served	70	Monday to Friday	10–5 pm	3564
5. Community centre	Lunch served	30	Monday to Friday	11:30–1:30 pm	4722

**Table 2:** Intake criteria required for the lowest and highest category scores (0 and 10 respectively). Dairy portion sizes retrieved from the Portion sizes: Food Fact Sheet. British Dietetic Association (BDA) [27].

Category	Category Score-0	Category Score-10
1. Free sugar intake	10% of total energy	<5% of total energy
2. Sodium intake	≥1.5 times recommended intake (7–10 years: ≥2953.75 mg 11–18 years: ≥3544.5 mg/d)	Sodium intake recommended by age group (7–10 years: ≤1969.17 mg11–18 years: ≤2363 mg/d)
3. Fat intake	≥45% of total energy	≤35% of total energy
4. Saturated fat intake	≥16% of total energy	≤11% of total energy
5. Protein intake	≤10% of total energy	≥14.5 and ≤15.5% of total energy
6. Carbohydrate intake	≤40% of total energy	≥50% of total energy
7. Dairy products	0 portions	3 portions (1 portion of milk: 200 mL, cheese: 30 g and yoghurt: 125 g)
8. Fibre intake	0 g/d	≥30 g/d
9. Processed meat	≥70 g/d	0 g/d
10. Water intake	≤3 glasses (600 g/d)	≥6 glasses (1200 g/d)
11. Fruit and vegetables	0 portions (0 g/d)	5+ portions (400 g/d)

**Table 3:** Elements of the School Foods Standards used to score lunch meal on an attending and a non-attending club day.

School Food Guidelines	Minimum Score	Maximum Score
*A. Starchy foods*		
1. One or more portions of food from this group every day	0	1
*B. Fruit and vegetables*		
1. One or more portions of vegetables or salad as an accompaniment every day	0	1
2. One or more portions of fruit every day	0	1
*C. Dairy and alternatives*		
1. A portion of food from this group every day	0	1
2. Lower fat milk must be available for drinking at least once a day during school hours	0	1
*D. Protein sources*		
1. A portion of food from this group every day	0	1
*E. Discretionary foods*		
1. No snacks, except nuts, seeds, vegetables, and fruit with no added salt, sugar, or fat		
2. No confectionery, chocolate, or chocolate-coated products	0	1
3. Desserts, cakes, and biscuits are allowed at lunchtime. They must not contain any confectionery	0	1
4. Salt must not be available to add to food after it has been cooked	0	1
5. Any condiments must be limited to sachets or portions of no more than 10 g or one teaspoonful	0	1
*F. Healthier drinks*		
1. One of the below permitted drinks Plain water (still or carbonated); Lower fat milk or lactose-reduced milk; Fruit or vegetable juice (max 150 mL); Plain soya, rice or oat drinks enriched with calcium; plain fermented milk (e.g., yoghurt) drinks; Combinations of fruit or vegetable juice with plain water (still or carbonated, with no added sugars or honey); Combinations of fruit juice and lower fat milk or plain yoghurt, plain soya, rice, or oat drinks enriched with calcium; cocoa and lower fat milk; flavoured lower fat milk, all with less than 5% added sugars or honey; Tea, coffee, or hot chocolate; Combination drinks are limited to a portion size of 330 mls; They may contain added vitamins or minerals, and no more than 150 mls of fruit or vegetable juice. Fruit or vegetable juice combination drinks must be at least 45% fruit or vegetable juice	0	1

Department for Education (2015). School Food Standards [14]. For each component where food consumed met the criterion, a score of 1 was given. If the element was not met, then a score of zero was given instead. Therefore, the maximal score for intake was 12 and the lowest possible score 0.

**Table 4:** The percentage of children meeting dietary recommendations on attending and non-attending days. Statistically significant differences (*p* < 0.05) are highlighted with *.

Diet Quality Category	Attending Club Day	Non-Attending Club Day
Carbohydrate *	71.9%	49.1%
Free sugars	8.8%	21.1%
Fat *	70.2%	49.1%
Saturated fat *	70.2%	38.6%
Dairy	0%	0%
Protein	5.3%	0%
Water	10.5%	10.5%
Processed meat	64.9%	61.4%
Fibre	1.8%	1.8%
Sodium	91.2%	87.8%
Fruit and vegetables	3.5%	10.5%
Total diet quality	0 %	0%

**Table 5:** The percentage of children meeting each assessable element of the School Food Standards. Statistically significant differences (*p* < 0.05) are highlighted with *.

	Starchy Foods (Total Score of 1) *	Fruit and Vegetables (Total Score of 2) *	Dairy and Alternatives (Total Score of 2)	Protein Sources (Total Score of 1) *	Discretionary Foods (Total Score of 5) *	Healthier Drinks (Total Score of 1)
Attending day	94.7%	31.6%	10.5%	91.2%	93%	38.6%
Non-attending day	73.7%	1.8%	10.5%	50.9%	59.6%	42.1%

## Data Availability

To request access to de-identified data, please contact the corresponding author. The data are not publicly available to prevent identification of participants.
